# Control of Moderate-to-Severe Plaque Psoriasis with Efalizumab: 24-Week, Open-Label, Phase IIIb/IV Latin American Study Results

**DOI:** 10.1111/j.1753-5174.2009.00024.x

**Published:** 2009-12

**Authors:** Fernando M Stengel, Valeria Petri, Gladys AM Campbell, Gladys Leon Dorantes, Magdalina López, Ricardo L Galimberti, Raúl P Valdez, Lucia F de Arruda, Mario Amaya Guerra, Edgardo N Chouela, Daiana Licu, International IMP25161 Study Group

**Affiliations:** * Centro de Educacion Médica e Investigaciones (CEMIC), Clinicas “Norberto Quirno”Buenos Aires, Argentina; † UNIFESP, Universidade Federal de São Paulo, Escola Paulista de MedicinaSão Paulo, Brazil; ‡ Hospital Universitário de BrasíliaBrasília, Brazil; § Hospital General de MexicoMexico City, Mexico; ¶ Hospital La RazaMexico City, Mexico; ** Hospital Italiano de Buenos AiresBuenos Aires City, Argentina; †† Hospital Universitario AustralPilar, Buenos Aires, Argentina; ‡‡ Pontificia Universidade Catolica de CampinasCampinas, Jundiai, Brazil; §§ Unidad Médica de Alta Especialidad N°25, IMSSMonterrey, NL, Mexico; ¶¶ Hospital General de Agudos “Dr Cosme Argerich”Buenos Aires, Argentina; *** Merck Serono SAGeneva, Switzerland

**Keywords:** Psoriasis, Efalizumab, Open-label, Phase IIIb/IV, Clinical Trial

## Abstract

**Introduction:**

Psoriasis is a debilitating, chronic inflammatory systemic disease affecting around 2% of the South American population. Biological therapies offer the possibility of long-term therapy with improved safety and efficacy.

**Methods:**

We conducted a multicentre, open-label, single-arm, Phase IIIb/IV study of adult patients (18–75 years) with moderate-to-severe plaque psoriasis who were candidates for systemic therapy or phototherapy. Patients received efalizumab subcutaneously (1.0 mg/kg/wk). The primary endpoint was the proportion of patients achieving a Physician Global Assessment (PGA) rating of “excellent” or “cleared” at Week 24. Safety outcomes were adverse events (AEs), serious AEs (SAEs) and abnormalities on laboratory tests.

**Results:**

Of 189 patients included in the intent-to-treat and safety populations, 104 (55.0%) were of Hispanic or Latino ethnicity. At Week 24, 92/189 (48.7%) patients achieved or maintained a PGA rating of “excellent” or “cleared”. AEs were reported by 161/189 (85.2%) patients, SAEs by 21/189 (11.1%). One patient died during the study (meningoencephalitis). Laboratory findings were consistent with previous experience.

**Conclusions:**

Efalizumab demonstrated sustained control of psoriasis up to 24 weeks in patients from Latin America, confirming results seen in Phase III studies conducted in North America and Europe.

## Introduction

Psoriasis is a T-cell-mediated, chronic inflammatory systemic disorder requiring long-term treatment. Conventional systemic therapies are associated with serious toxicities that can limit long-term use [1]. Efalizumab is a recombinant, humanized, monoclonal immunoglobulin G1 antibody that exerts antipsoriasis effects by blocking T-cell-dependent functions mediated by leucocyte function-associated antigen-1 [2]. Its efficacy and safety have been investigated in a clinical development programme based in Europe and North America that included over 2800 patients with moderate-to-severe psoriasis [3–10]. The open-label, Phase IIIb/IV study discussed here was performed to collect confirmatory data on the efficacy and safety of efalizumab in a previously unreported Latin American population.

## Methods

This was a 24-week, open-label, single-arm Phase IIIb/IV study (protocol IMP25161; ClinicalTrials.gov registration NCT00287118) conducted in 23 centres in Argentina, Brazil and Mexico between October 2004 and May 2006. Patients aged 18–75 years with moderate-to-severe plaque psoriasis who were candidates for systemic therapy or phototherapy were eligible. The extent of psoriasis had to involve at least 10% of total body surface area. Discontinuation of any systemic psoriasis treatment was required prior to commencement of the trial; in the case of biologics, a 3-month washout period was required. For women of childbearing potential and for men whose partners could become pregnant, consent to use an acceptable method of contraception and agreement to continue to practise an acceptable method of contraception for the duration of their participation in the trial and up to 3 months after the last dose of efalizumab, were mandatory for study participation. In addition, treatment regimens of β-blockers, angiotensin-converting enzyme inhibitors, antimalarial drugs, quinidine, interferon, or lithium had to be stable for at least 28 days prior to the first dose of trial medication. Key exclusion criteria included guttate, erythrodermic or pustular psoriasis as the sole or predominant form of psoriasis. Patients were also ineligible if they had active disease rebound during or following discontinuation of previous efalizumab treatment (i.e. a Psoriasis Area and Severity Index [PASI] > 125% from baseline and/or new predominant morphology of psoriasis) if this outcome was related to efalizumab adverse events (AEs) or to lack of efalizumab efficacy; however, patients were eligible for study drug medication if active disease rebound was due to a nondrug reason (e.g. infection or vaccination). Other exclusion criteria were as follows: history of severe allergic or anaphylactic reactions to humanised monoclonal antibodies; history of or ongoing uncontrolled infection; seropositivity for HIV, hepatitis B or C virus; pregnancy or lactation; white blood cell count <4 × 10^9^/L or >14 × 10^9^/L; history of clinically significant thrombocytopenia, bleeding disorders or a platelet count <100 × 10^9^ cells/L; history of active tuberculosis; presence of malignancy within the past 5 years (except fully resolved basal cell or squamous cell skin cancer); hepatic cirrhosis; serum creatinine >2 times the upper limit of normal; hospital admission for cardiac disease, stroke, or pulmonary disease within the last year; history of substance abuse within the last 5 years; and any medical condition that, in the judgement of the investigator, would jeopardize the subject's safety following exposure to study drug. The study complied with the Declaration of Helsinki and Good Clinical Practice guidelines, with approval by the independent ethics committee/institutional review board for each country. Patients gave written informed consent.

Participants underwent initial screening 14 days before the first efalizumab injection. Discontinuation of any systemic psoriasis treatment was mandatory before starting study medication; no washout was required. All participants received efalizumab administered subcutaneously, starting with an initial conditioning dose of 0.7 mg/kg at baseline (study Day 0), followed by 23 weekly doses of 1.0 mg/kg.

Participants were evaluated on study Day 0 and at Weeks 2, 4, 8, 12 and 24 (or at early treatment termination), with a follow-up visit at Week 32. Clinical efficacy assessments performed at all visits included the dynamic Physician Global Assessment (PGA) and the PASI. Quality of life (QoL) was assessed at all visits using the Medical Outcomes Study Short-Form (36-Item) Health Survey (SF-36) and the Dermatology Life Quality Index (DLQI).

The PGA measured the global response of all psoriatic lesions to therapy by comparing the subject's present condition to baseline photographs or body diagrams. The assessor classified response by considering erythema, scaling, plaque thickness and percentage of body surface area affected. Ratings of cleared and excellent represented a 100% improvement (remission) and 75% to 99% improvement of all clinical signs and symptoms relative to baseline, respectively. The PASI determines the extent of cutaneous psoriasis by dividing the body into 4 anatomical regions (i.e. head, trunk, upper limbs, and lower limbs) and, for each region, grading the severity of erythema, induration/thickness and scaling from 0 for “none” to 4 for “very severe”. The percentage of the region affected by disease (graded from 0 for “none” to 6 for 90% to 100% involvement, is also determined. A numerical score is derived that evaluates the severity of symptoms in terms of the total body surface area affected: this score can range from 0 to 72, with higher scores indicating more severe disease. The DLQI consists of 10 questions concerning the impact of skin disease and its treatment on a subject's life over the past week. These questions relate to physical discomfort, emotional distress, work and leisure activities, family and social relationships, sexuality and treatment burden. Each question is posed such that possible responses are “very much”, “a lot”, “a little” or “not at all”; patients can also indicate that particular questions are not relevant. DLQI scores range from 0 to 30, with higher scores indicating greater impact of disease (i.e. poorer QoL).

Safety assessments, which included treatment-emergent AEs, serious AEs (SAEs) and AEs leading to permanent treatment discontinuation, were coded for analysis using MedDRA. Summaries of AEs were prepared by MedDRA System Organ Class and Preferred Term for all events, the most frequent events, all events by severity, all events by relation to trial medication, all SAEs and all AEs leading to withdrawal from treatment. The severity of adverse events was assessed as mild (the patient was aware of the event or symptom but it was easily tolerated), moderate (the patient experienced sufficient discomfort to interfere with, or reduce, his or her usual level of activity), severe (the patient experienced a significant impairment of functioning and was unable to carry out his or her usual activities) or very severe (the patient's life was at risk from the event). A serious adverse event was defined as an event resulting in death or that was life-threatening, required hospitalization or prolongation of existing hospitalization, resulted in persistent or significant disability or incapacity, was a congenital anomaly or birth defect, or was a medically important condition (the event did not have to be immediately life-threatening or result in death or hospitalization, but was clearly of major clinical significance). Routine clinical laboratory evaluations comprised haematology (i.e. red blood cell count, haemoglobin, haematocrit, platelet count, white blood cell count and white blood cell differential) and biochemistry tests (i.e. urea and electrolytes, liver function, blood urea nitrogen, blood glucose, creatine phosphokinase, and uric acid) performed at baseline and at Weeks 2, 12 and 24 (or at early treatment termination). Platelet counts were also performed at Weeks 4 and 8.

### Analysis Populations

The following populations were defined for analysis:

- Intent-to-treat (ITT) and safety population: patients who received ≥1 dose of trial medication.
- Per-protocol (PP): all patients who did not have major protocol violations. The PP population was used for supportive analyses of the primary and secondary endpoints.

### Outcome Measures

The primary efficacy endpoint was the proportion of patients with a PGA score of “excellent” or “cleared” at Week 24 (ITT and PP populations). The secondary endpoint was the proportion of patients achieving “at least good disease control” at Week 24, defined as a PASI score of <8 or a 50% reduction from baseline in PASI score (PASI-50), a DLQI score of <6, and no SAEs, treatment-related SAEs, or early withdrawal (ITT and PP populations). This endpoint provides a comprehensive assessment of benefit–risk ratio and takes into account the multidimensional aspects of psoriasis. It is similar to the “safe psoriasis control” measure proposed in recent publications [11,12]. The disease control endpoint was also evaluated in a sensitivity analysis, which excluded the PASI-50 measure from the composite endpoint (i.e. the only PASI requirement was a score of <8).

Tertiary endpoints were the proportion of patients achieving a ≥75% reduction from baseline in PASI score (PASI-75) and PASI-50; changes in DLQI and SF-36 scores; and changes in scores for the PASI, Nail Psoriasis Severity Index, Palmoplantar Pustular Psoriasis Area and Severity Index, and Psoriasis Scalp Severity Index (ITT population). Results of the nail, scalp and palmoplantar psoriasis assessments are reported elsewhere [13].

Analyses of efficacy in the ITT population were performed using the last observation carried forward (LOCF) approach. The primary endpoint was also analysed using the nonresponder imputation technique, in which all patients with missing data at Week 24 were counted as nonresponders. No formal hypothesis testing or adjustments were performed. It was estimated that 220 patients would have to be screened to yield a sample of sufficient size to accurately assess efalizumab's safety and efficacy profile in this target population.

## Results

Between October 2004 and September 2005, 220 patients were screened, of whom 31 patients were considered screening failures, mostly for reasons related to laboratory results. Thus, 189 were enrolled and received treatment (ITT and safety populations). A total of 137 patients (72.5%) completed the 24-week treatment period. The main reasons for discontinuation from the treatment period were AEs in 30 patients (57.7%), lack of efficacy in 11 patients (21.2%), and protocol violation by 2 patients (3.8%). The PP population comprised 124 patients.

The median age of patients in the ITT population was 46 years (range 19–74 years), two-thirds were men, and 55% were of Hispanic or Latino ethnicity (Table 1). The mean ± standard deviation (SD) PASI score at baseline was 24 ± 9.5 and 59.0% had a baseline PASI score of ≥20 points. Reported medications at baseline were typical of a psoriasis population, with nonsteroidal anti-inflammatory drugs, analgesics and topical psoriasis medications frequently used. The mean (±SD) time on treatment was 139 ± 45 days. Almost two-thirds of patients (64.5% [122/189]) received all 24 planned weekly injections.

**Table 1:** Baseline demographic and disease characteristics of the intent-to-treat population (N = 189)

Characteristic	Value
Median age (range), years	46 (19–74)
Male, n (%)	134 (70.9)
Race, n (%)	
White	125 (66.1)
Black	7 (3.7)
Asian	0
Other	57 (30.2)
Hispanic or Latino ethnicity, n (%)	104 (55.0)
Median weight (range), kg	80 (46–120)
Median body mass index (range), kg/m^2^*	28.7 (16.5–45.3)
Median duration of psoriasis (range), years*	15 (1–46)
Patients with prior psoriasis therapy, n (%)	158 (83.6)
Patients with prior systemic therapy, n (%)*	153 (81.0)
Median PASI score (range)†	22 (7–61)
PASI score ≥20, n (%)‡	111 (59.0)
Median DLQI score (range)§	11.5 (0–29)
Median SF-36 score (range)	
Total score¶	550.3 (66.4–778.5)
Mental component score§	262.5 (28.0–396.0)
Physical component score**	294.4 (21.7–395.0)

* Psoralen ultraviolet A therapy, ciclosporin, systemic corticosteroids, methotrexate, systemic retinoids, mycophenolate mofetil, thioguanine, hydroxyurea, sirolimus, azathioprine and 6-mercaptopurine;

† N = 180;

‡ N = 188;

§ N = 180;

¶ N = 177;

** N = 179.

DLQI, Dermatology Life Quality Index; PASI, Psoriasis Area and Severity Index; SF-36, Medical Outcomes Study Short-Form (36-Item) Health Survey.

### Endpoints

**Figure 1:**
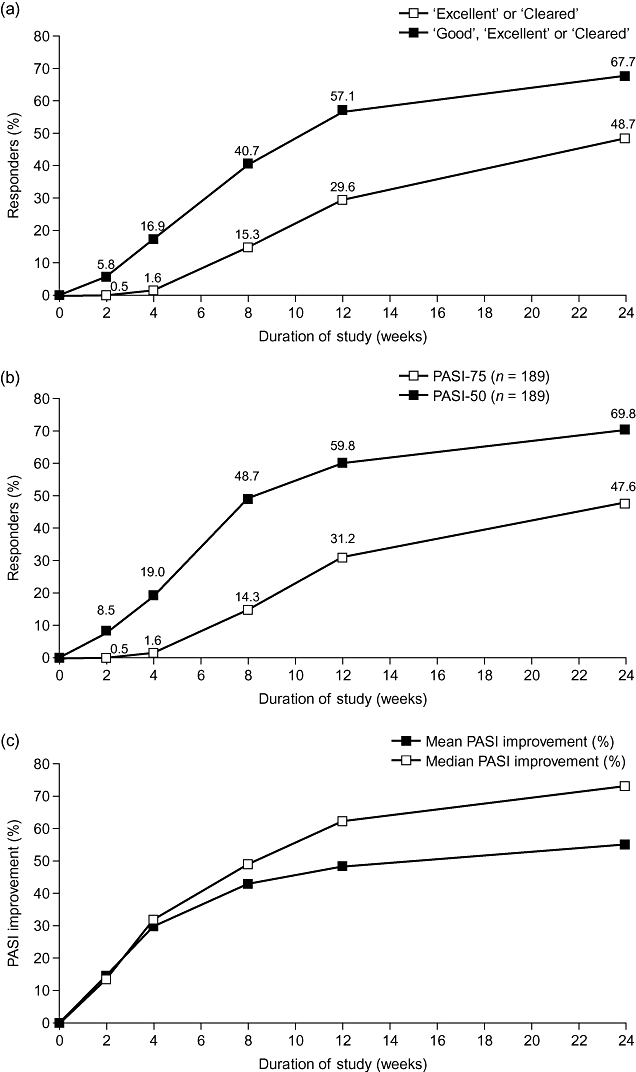
(a) Physician Global Assessment score over time during treatment with efalizumab (intent-to-treat population, last observation carried forward; N = 189). (b) Proportion of responders (95% CI) achieving a ≥75% reduction from baseline in Psoriasis Area and Severity Index (PASI) score (PASI-75) and a ≥50% reduction from baseline in PASI score (PASI-50) over time during treatment with efalizumab (intent-to-treat population, last observation carried forward). (c) Mean and median (95% CI) percentage improvement in PASI score over time during treatment with efalizumab (intent-to-treat population, last observation carried forward).

In the ITT population, 48.7% (92/189) of patients achieved or maintained a PGA score of “excellent” or “cleared” (95% confidence interval [CI] 41.6–55.8%; Fig. 1a) at Week 24 (nonresponder imputation analysis 46.0% [87/189]; 95% CI 38.9–53.1%); in the PP population this rate was 65.3% (81/124; 95% CI 56.9–73.7%). At Week 24, 67.7% (128/189) in the ITT population achieved or maintained a PGA score of “good” or better (Fig. 1a).

In the ITT population, 47.1% (89/189) achieved “at least good disease control” at Week 24 (95% CI 40.0–54.2%); in the PP population this rate was 65.3% (81/124; 95% CI 56.9–73.7%). “Safe disease control” at Week 24 was achieved by 50.0% (62/124; 95% CI 41.2–58.8%) in the PP population and 36.0% (68/189; 95% CI 29.1–42.8%) in the sensitivity analysis.

At Week 24, 90 patients (47.6%; 95% CI 40.5–54.7%) were classified as PASI-75 responders and 132 (69.8%; 95% CI 63.3–76.4%) were classified as PASI-50 responders (Fig. 1b).

Median PASI and DLQI scores with respect to study visit are summarized in Table 2. Percentage change from baseline in PASI score improved by visit (Fig. 1c). At Week 12, the median percentage change from baseline was 61.9% (interquartile range 34–79%) and the mean ± SD was 49.0% ± 44.0%; at Week 24, using LOCF, the median percentage change from baseline had increased to 72.8% (95% CI 65.3–78.6%) and the mean ± SD had increased to 55.2% ± 48.5%. At Week 24, the median improvement in DLQI score (N = 180) was 6 (95% CI 5–7). The median improvements in SF-36 total (N = 177), physical (N = 179) and mental (N = 180) scores were 35.5 (95% CI 21.4–57.3), 17.2 (95% CI 9.7–23.6) and 22.5 (95% CI 11.3–32.0), respectively.

**Table 2:** Median Psoriasis Area and Severity Index (PASI) and Dermatology Life Quality Index (DLQI) scores over the duration of the 24-week treatment period in the intention-to-treat population using last observation carried forward analysis

Visit	Median PASI score (interquartile range)	Median DLQI score (interquartile range)
Baseline	21.9 (16–29)	11.5 (7–16)
Week 2	17.2 (13–26)	8.0 (4–12)
Week 4	14.5 (11–20)	7.0 (3–11)
Week 8	10.4 (7–16)	5.0 (1–9)
Week 12	7.9 (5–15)	4.0 (1–9)
Week 24	5.8 (2–14)	3.0 (1–9)

### Safety and Tolerability

During the treatment phase, 161/189 (85.2%) patients reported at least one AE; 152 (80.4%) experienced at least one AE during Weeks 0–12 vs 67 (35.4%) during Weeks 13–24. In 125 patients (66.1%), at least one AE was considered possibly or probably related to efalizumab.

The most frequently reported treatment-emergent AEs, particularly during the first 12 weeks, were consistent with the syndrome of “acute AEs” (influenza-like symptoms such as headache, fever, chills, myalgia, nausea or vomiting within 48 hours of administration) (Table 3). Post-treatment AEs were reported in 69 patients (36.5%), 20 of whom continued with efalizumab after completing the study treatment period. Thirty-one patients (16.4%) experienced a total of 46 treatment-emergent AEs that led to withdrawal of efalizumab; psoriasis (6.3%) and arthritis (2.1%) were the most common AEs cited.

**Table 3:** Summary of the most frequent treatment-emergent adverse events (TEAEs) (reported in ≥5% of patients) in the safety population (N = 189)

	Number of patients (%)	
Adverse events	Weeks 1–12	Weeks 13–24
Total	152 (80.4)	67 (35.4)
Serious TEAEs	15 (7.9)	8 (4.2)
Severe or very severe TEAEs	29 (15.3)	16 (8.5)
Discontinued because of a TEAE	21 (11.1)	11 (5.8)
TEAE term		
Headache	68 (36.0)	6 (3.2)
Arthralgia	23 (12.2)	6 (3.2)
Myalgia	21 (11.1)	7 (3.7)
Influenza-like illness	20 (10.6)	3 (1.6)
Pyrexia	20 (10.6)	3 (1.6)
Chills	18 (9.5)	0
Back pain	13 (6.9)	5 (2.6)
Diarrhoea	13 (6.9)	1 (0.5)
Nausea	13 (6.9)	1 (0.5)
Influenza	12 (6.3)	2 (1.1)
Psoriasis	10 (5.3)	8 (4.2)

SAEs were reported in 21 patients (11.1%) and included psoriasis, arthritis, arthralgia and myalgia. Post-treatment SAEs were reported in 22 patients (11.6%). Treatment-emergent musculoskeletal and connective tissue disorders (most commonly arthralgia, myalgia, back pain, and arthritis) were reported in 71 patients (37.6%). One patient died during the study from meningoencephalitis probably of bacterial origin. Due to the mechanism of action of efalizumab, it could not be excluded that efalizumab played a role in the evolution of the infection. Five infection-related SAEs occurred in four patients during the treatment period of the study (cytomegalovirus infection, meningitis aseptic, pneumonia, tooth abscess and sepsis [the tooth abscess and sepsis were reported in the subject who died]). The pneumonia, tooth abscess and sepsis were considered to be probably related to the trial medication and the cytomegalovirus infection and meningitis aseptic to be possibly related. During the post-treatment period, pneumonia was reported in the patient who died, which was also considered to be probably related to the trial medication.

Total white blood cell, lymphocyte and neutrophil counts increased during treatment (Table 4). For most patients the maximum toxicity was grade ≤1. At baseline, none of the 53 patients with evaluable blood samples tested positive for binding antibodies to efalizumab. At Week 32, 9/96 (9.4%) patients tested positive for binding antibodies to efalizumab.

**Table 4:** Median (range) clinical laboratory values in the safety population

	Visit					
	Baseline		Week 12		Week 24	
Parameter (unit)	No. pts	Value	No. pts	Value	No. pts	Value
Red blood cell count (×10^12^/L)	186	5.0	180	5.0	155	5.0
		(3.8–6.9)		(3.4–6.9)		(3.6–6.1)
Haemoglobin (g/L)	186	151	180	150	155	150
		(114–202)		(103–190)		(99–189)
Haematocrit	186	0.45	180	0.45	155	0.44
		(0.36–0.61)		(0.32–0.62)		(0.35–0.55)
White blood cell count (×10^9^/L)	186	7.0	180	10.0	154	10.2
		(3.8–14.5)		(5.8–24.7)		(4.5–17.6)
Lymphocytes (×10^9^/L)	186	1.8	180	4.2	154	4.0
		(0.7–3.7)		(1.4–10.3)		(0.7–9.7)
Neutrophils (×10^9^/L)	186	4.4	180	5.0	154	4.9
		(1.6–12.4)		(1.6–20.5)		(1.5–12.7)
Platelets (×10^9^/L)	186	249	169	240	155	241
		(127–556)		(82–3125)		(105–590)
Sodium (mmol/L)	186	140	181	140	152	141
		(133–148)		(134–147)		(131–148)
Potassium (mmol/L)	185	4.3	181	4.3	152	4.4
		(3.5–5.1)		(3.7–5.6)		(3.5–13.6)
Blood urea nitrogen (mg/dL)	176	13.7	139	14.0	104	13.6
		(4.7–32.0)		(5.3–24.3)		(5.0–23.9)
Creatinine (µmol/L)	186	78	180	77	151	79
		(34–143)		(33–161)		(41–138)
Total bilirubin (µmol/L)	186	9.6	180	8.3	151	8.4
		(2.9–32.1)		(2.6–35.2)		(3.2–37.6)
Blood glucose (mmol/L)	186	5.1	180	5.2	152	5.2
		(3.4–17.7)		(3.0–15.5)		(3.6–23.8)
Alanine aminotransferase (U/L)	185	29.0	181	26.0	152	26.5
		(6.0–102.0)		(7.0–241.0)		(8.0–122.0)
Aspartate aminotransferase (U/L)	185	23.0	181	23.0	151	23.0
		(11.0–69.0)		(10.0–109.0)		(10.0–123.0)
Alkaline phosphatase (U/L)	186	80.5	181	85.0	151	86.0
		(39.0–189.0)		(32.0–244.0)		(39.0–298.0)
Lactate dehydrogenase (U/L)	185	187.0	181	172.0	152	167.5
		(76.0–398.0)		(152.0–200.0)		(86.0–353.0)
Creatine phosphokinase (U/L)	186	100.5	181	92.0	152	87.5
		(17.0–1369.0)		(16.0–470.0)		(19.0–1528.0)
Uric acid (×10^9^/L)	186	364.0	181	375.0	152	356.0
		(149.0–690.0)		(156.0–714.0)		(153.0–684.0)

## Discussion

In this study, 48.7% of patients reached a PGA rating of at least “excellent” at 24 weeks (ITT LOCF analysis) and 47.6% were classified as PASI-75 responders at this timepoint, consistent with rates reported in European and North American populations [5,6,10]. These findings, however, should be interpreted while considering the main study limitations, which were the open-label design and the lack of placebo arm.

We used an additional measure of “at least good disease control” to determine outcome. As for the “safe psoriasis control” measure proposed by Papp and Henninger [11,12], this endpoint reflects improvements not only in the clinical severity of lesions but also in QoL and an assessment of the tolerability of treatment. By this measure, nearly half of the patients studied derived important benefit from efalizumab treatment without incurring major safety concerns.

The safety profile for efalizumab was also acceptable and consistent with previous results [3–10]; no new safety concerns were identified. AEs occurred most frequently during the first 12 weeks of treatment, the most common events being consistent with the syndrome of influenza-like symptoms or acute AEs that are known to occur with initial efalizumab treatment. Similar to previous trials, the SAEs and AEs most commonly leading to treatment discontinuation were related to exacerbation of psoriasis and/or arthritis [3–10]. Laboratory findings were also consistent with previous experience. Treatment-emergent musculoskeletal and connective tissue disorders were seen in 37.6% of patients, and long-term data have indicated that the incidence of these AEs remains stable for up to 3 years of continuous efalizumab treatment [13].

Although results from the present study indicate that efalizumab has an acceptable safety profile in patients with psoriasis, opportunistic infections have been reported in post-marketing surveillance. In particular, instances of progressive multifocal leucoencephalopathy due to JC virus infection have been observed in patients receiving efalizumab continuously for more than 3 years. After evaluation of all available safety data, the European Medicines Agency has concluded that the benefits of efalizumab therapy no longer outweigh the associated risks and have recommended suspension of marketing authorization (as of 19 February 2009). In addition, efalizumab has been voluntarily withdrawn from the US market.

To our knowledge, the present study is the only prospective trial confirming the efficacy and safety of efalizumab in a Latin American population with moderate-to-severe psoriasis.
